# How supranational institutions benefit from crises: Member states’ solidarity and the EU's image during the COVID-19 pandemic

**DOI:** 10.1177/14651165231156846

**Published:** 2023-03-03

**Authors:** Achillefs Papageorgiou, Waltteri Immonen

**Affiliations:** Political Science, 3835 University of Helsinki, Finland; Political Science, 8058 University of Turku, Finland

**Keywords:** COVID-19 pandemic, EU image, Eurobarometer, institutional solidarity, interstate solidarity

## Abstract

In this article, we demonstrate how solidarity between member states can have a positive effect on the image of the EU, even if the latter's actions in handling a crisis such as the COVID-19 pandemic are deemed unsatisfactory. Employing data from a special Eurobarometer survey enriched with data from the Oxford's COVID-19 government response tracker, we show that European citizens who are more satisfied with interstate solidarity have to a greater extent a positive image of the EU compared to citizens who are less satisfied. We also show that this effect is further pronounced in the case of EU citizens who are less satisfied with institutional solidarity, which is the solidarity going from EU institutions to the member states.

## Introduction

Major crises can serve as turning points that either wreck or reinforce the image of supranational institutions. On the 24 January 2020, the first European case of COVID-19 was reported in France and, by mid-March 2020, the World Health Organization had declared Europe the epicenter of the pandemic. Solidarity is defined as a “set of feelings of belonging together” (Parijs, 2004: 375) that affects both attitudes and expressions of mutual cooperation (Banting and Kymlicka, 2017). It plays a key role in holding together complex democratic polities (Ferrera and Burelli, 2019) and is mentioned in various European Union (EU) treaties (Reinl, 2022).

Interstate solidarity within the EU, refers to mutual aid and cooperation between member states. It evolved at the onset of the COVID-19 pandemic through the dispatching of medical supplies and collaboration in treating patients. Interstate solidarity was not without its challenges (e.g., competition between member states for medical supplies), but member states soon enough realized the need for collaboration in fighting the pandemic. The main reason for this, was that all member states were affected by the pandemic and no single EU country could be blamed for causing it (Haverland et al., 2022). This was not the case in either the Eurozone or the so-called refugee crisis that affected the member states to different degrees. As previous research has demonstrated, exogenous shocks, such as pandemics (Katsanidou et al., 2022), are more likely to increase support for interstate solidarity than home-grown crises (Genschel et al., 2021).

At the supranational level, the EU also adopted a series of measures geared towards containing the spread of the pandemic and accelerating the recovery process of its member states, e.g. the Next Generation EU (NGEU) recovery package. After hard negotiations (Tesche, 2022), all member states agreed to depart from the logic of previous rescue packages, which typically consisted of government-backed loans under strict conditions of fiscal consolidation (de la Porte and Jensen, 2021). The fact that the NGEU package was financed by a collective borrowing from the markets marked an unprecedented act of solidarity (Christie et al., 2021). In this study, we refer to this type of solidarity—measures adopted by the EU to assist its member states—using the term institutional solidarity.

Previous relevant literature has focused on uncovering the factors that trigger solidarity during a crisis (Haverland et al., 2022; Unger et al., 2023). For example, Haverland et al. (2022) argued that citizens who experience the health and social effects of the pandemic (but not the financial effects) are more likely to support expressions of solidarity such as fiscal transfers. In a recent publication, Unger et al. (2023) showed that self-interest, justice attitudes and general support of European integration explain the public's support for redistributive EU measures in Austria, Germany and Italy. In this article, we study the consequences of solidarity between countries for the image of an institution of which they are members. More precisely, we examine the extent to which the image of the EU benefits from perceived solidarity between its member states in times of a common threat such as the COVID-19 pandemic. In the Eurobarometer survey, the concept “image of the EU” is captured by the question “In general does the EU conjure up for you a very positive, fairly positive, neutral, fairly negative or very negative image?”. The question is also relevant to the concept of Euroscepticism/Europhilia, which expresses doubt/support towards European integration.

Do citizens who are satisfied with interstate solidarity in fighting the pandemic hold a more positive view of the EU compared to those citizens who are less satisfied with interstate solidarity? Because the EU is a community of diverse countries, we initially expect that any kind of solidarity also contributes to a positive image of the organization to which they belong. We also seek to clarify how satisfaction with the two types of solidarity—interstate and institutional (i.e., measures taken by the EU to support its member states)—in fighting the pandemic interact in shaping a negative or a positive image of the EU. To the best of our knowledge, this is the first article to examine this issue and it contributes to a recent warranted research call (Haverland et al., 2022) for further investigation of the new EU mechanisms of public support, such as the NGEU.

Employing data from a special Eurobarometer survey and checking for the robustness of our findings, we find that satisfaction with interstate solidarity encourages a positive effect on the image of the EU. Our study also demonstrates that the effect of satisfaction with interstate solidarity on the image of the EU was more pronounced in the case of Czech Republic, the country most severely impacted by the pandemic proportional to its population during the period of the Eurobarometer survey (March and April 2021). Lastly, we show that the effect of satisfaction with interstate solidarity on the image of the EU is stronger for those citizens who are less satisfied with EU measures in fighting the pandemic compared to citizens who are more satisfied. From the EU's perspective, this finding underscores the benefits that an institution enjoys when promoting solidarity between its members, especially in times of a crisis where many citizens might not be satisfied by the way in which supranational institutions handle critical issues. Namely, in times of a crisis, the image of a supranational organization such as the EU benefits from solidarity between its member states, as interstate solidarity fills the lacuna left over from institutional solidarity.

## EU measures in fighting the COVID-19 pandemic: institutional solidarity

In this section, we attempt to present a short but concise overview of the measures consisting institutional solidarity, meaning vertical measures coordinated by the EU and channeled through the European Commission (EC), the European Central Bank (ECB) and the European Investment Bank (EIB), towards assisting member states fighting the pandemic (for a more detailed analysis see Tesche, 2022).

It should be clear that using a positive term such as solidarity does not imply that European citizens are necessarily satisfied with the measures adopted by the EU in fighting the pandemic. More precisely, almost half of the respondents participating in the Eurobarometer survey stated that they are not satisfied (see the Online appendix). We use the term solidarity here to underline that the EU considers these measures as a means of assisting member states to recover from the pandemic. For example, both the European Council and the EC referred to the EU measures against the pandemic as “European solidarity in action” (European Council, 2021; European Commission, 2020). Goldberg et al. (2021), emphasized the fact that most of the EU measures aimed at promoting the financial recovery of member states labeled this type of solidarity as “fiscal solidarity”. We prefer to use the term institutional solidarity, as a means of demarcating it from interstate solidarity (solidarity between member states). Alongside measures aimed towards containing the spread of the virus, the EU also adopted a series of recovery actions, meaning measures aimed towards mitigating the financial fallout caused by the pandemic. These measures were mainly channeled through the EC, the ECB and the EIB.

The EC—through *SURE* (*S*upport to mitigate *U*nemployment *R*isks in an *E*mergency)—provided loans of up to a hundred billion euros to member states towards reducing unemployment and mitigating the sudden increase of public expenditure caused by the pandemic. Adopting specific instruments that support member states in fighting unemployment in times of crisis is not a novel concept and has its origins in the European sovereign debt crisis (henceforth euro crisis). Solidarity in *SURE* was expressed through a mechanism where member states provided loan guarantees to other member states (Lindner, 2022) and the EC issued social bonds for the market to alleviate the negative economic effects engendered by the pandemic (Cicchiello et al., 2022).

The ECB assisted lending through a €1850b pandemic emergency purchase program by freezing key interest rates at historically low levels and by relaxing the standards for collaterals when borrowing directly from the ECB (Spielberger, 2022). Also, in response to the financial upheavals caused by the pandemic, the ECB bought bonds directly from banks, thus increasing the funds that could be distributed to both households and companies through affordable loans. Further, the EIB created a European guarantee fund that supported small- and medium-sized companies (SMEs) that struggled with the economic impact of the pandemic (Howarth et al., 2020). According to the president of the EIB, Werner Hoyer, targeting half of the 2021 fund in SMEs is important as “Europe and European citizens [should] see solidarity in different forms” (European Investment Bank, 2022).

Clearly, the most ambitious expression of solidarity by the EU was to reduce the burden especially of those member states worst hit by the pandemic. The NGEU planned on distributing €750b (Rhodes, 2021), €360b in loans and €390b in grants on the basis of “implementation of country-specific recovery and resilience plans” (Tesche, 2022: 11). Member states needed to submit their national recovery and resilience plans, which were first assessed by the EC and then approved by the Council of the EU (Council) (European Commission, 2021). An important element of the NGEU recovery package is that the biggest proportion of the financial assistance is administered as grants and not as loans, as was the norm in previous crises such as the euro crisis (Armingeon et al., 2022; Katsanidou and Otjes, 2016). The vast amount of grants and loans are administered through the Recovery and Resilience Fund (Theodoropoulou, 2022), which is a temporary recovery instrument that finances member states from the onset of the pandemic until the end of 2026. Although the adoption of grants was in line with the demands of southern European countries (such as Italy, France and Spain), the NGEU nevertheless refrained from mutualizing public debt, an idea that was pointedly rejected by Austria, Denmark, the Netherlands, Sweden, Germany and Finland. Yet, despite the riff in opinions between different member states on issues like the Corona bonds or the amount of grants to be distributed as a proportion of total financial assistance, the final adoption of the NGEU rescue package was an act of solidarity aimed at assisting financially the member states worst hit by the pandemic. As Jones (2021: 2) puts it, the NGEU “underscores the breadth of solidarity across EU member states, it consolidates an expansion of competences within the European Commission, and it lays the foundations for more effective pan-European macroeconomic stabilization”.

### Interstate solidarity within the EU

Contrary to institutional solidarity, which is vertically coordinated from the supranational level, interstate solidarity within the EU is horizontal as it concerns the collaboration and mutual aid between member states (Katsanidou et al., 2022). It is important to note that when asked to indicate their level of satisfaction with both types of solidarity, the extent to which European citizens are able to distinguish between actions of institutional and interstate solidarity is not always clear. For example, it is possible that citizens of some of the members states that contribute the most to the EU budget might perceive certain vertical actions of institutional solidarity as another form of interstate solidarity channeled through EU mechanisms. Nevertheless, we believe that the clear formation of the questions regarding interstate and institutional solidarity in the Eurobarometer survey helps respondents to distinguish between the two. Interstate solidarity is asked with the question “How satisfied are you with the solidarity between EU member states in fighting the Corona virus pandemic?” whereas institutional solidarity with the question “In general how satisfied are you with the measures^1^ taken to fight the coronavirus pandemic by the European Union”.

Interstate solidarity raises the issue of impelling members to act against their own self-interest, as solidarity entails among others a certain financial cost for those exercising it. This issue is especially relevant within the European context as the EU is a diverse, still evolving union of countries where “the long-term effects of integration on growth and problem-solving capacity are uncertain, and […] the effects, both positive and negative, will be unevenly distributed among member states” (Schelkle, 2022: 5). The quest for interstate solidarity in the case of a global crisis such as the pandemic becomes even more perplexed considering that it might conflict with imperatives for national solidarity (solidarity between citizens belonging to the same member state). This begs the question: Why should citizens of a member state agree to send medical equipment to another member state, when their own country is being threatened by the same crisis?

The idea that solidarity between member states is built on the premise of a European identity that is common to a European *Demos* is bounded by the fact that there is no single European *Demos*, nor a desire to construct one (Nicolaïdis, 2003; 2013). Conversely, within the EU there exist many *Demoi*, with different needs that should be respected in the making of a European Demoicracy (Nicolaïdis, 2013). Thus, we do not mean that European solidarity is entirely stripped from an emotional and cognitive element of group belonging (Tajfel, 1981) and therefore an implicit or explicit referral to out-groups, as in who counts as “us” and “them” (Tuomela, 2013: 243).

We argue that interstate solidarity within the EU can thrive only through a mutual respect of different polities and “their respective pasts, their social pacts, their political systems, their cultural traditions, their democratic practices” (Nicolaïdis, 2012: 248). In that sense, solidarity between member states is an inherent element of belonging to a trans-national union such as the EU (Nicolaïdis, 2013), where satisfaction with interstate solidarity is channeled into a positive image of the EU itself. The EU as a Demoicracy is “neither a union of democratic states as ‘sovereignists’ or ‘intergovernmentalists’ would have it, nor a Union-as-a-democratic state to be, as ‘federalists’ would have it” (Nicolaïdis, 2013: 353); rather, it combines characteristics from both the supranational and national civic. This means that institutional and interstate solidarity interact with one another in shaping the EU image. Since the EU as a Demoicracy seeks to correspond to the different needs of its members, interstate solidarity is expected to be proportional to the negative effects of the pandemic's impacts on each member state. As Genschel and Jachtenfuchs (2021: 351) argued “expanded expectations of community to the transnational level as empathy with the worst affected member states led to vocal calls for more EU solidarity and leadership”. Empathy with a member state worst hit by a crisis accentuates the feeling of deservingness (Petersen, 2012) insofar as this member state is not blamed for causing the crisis. This is the case with the pandemic that was largely perceived as “a symmetric external shock, for which no country bears responsibility, but whose negative consequences are endured by all” (Wilmès et al., 2020).

The more the members of this union of different polities benefit from solidarity, the more willing they are to sustain and advance, as well as defend the group, against (possible or actual) threats or setbacks, even at the expense of their own self-interest (Khushf, 1999: 65). This creates the basis for a moral framework required in solidarity, which reveals and specifies the obligations of reciprocity within and among groups. The framework is indeed moral instead of, e.g., a legal one as the group is bound together by goodwill rather than fear of repercussions if they do not behave in a united manner (Klamert, 2022). Seen this way, solidarity between EU member states has this type of a positive moral framework where the emphasis is on the support and encouragement of beneficial actions rather than prohibiting or sanctioning unbeneficial ones. This is not to go so far as to claim that interstate solidarity is always “a fair procedure so that any principles agreed to are just” (Schelkle, 2022: 4), but rather the belief in a sort of togetherness, especially fellowship in shared experience. This creates an almost self-perpetuating circle whereby the interconnectedness of the group enhances mutual experience even further, thus adding to solidarity. It is also important to keep in mind that mere positive prudence is not enough to explain the whole of solidarity, as negative prudence must be also taken into consideration. In a situation where there is sufficient reason to expect solidarity, its lack thereof is “morally disapproved of” being not only imprudent but “shabby and reprehensible” as well (Bayertz, 1999: 18). Expectations of reciprocity make solidarity between member states interest-based as it is advisable for members to participate in mechanism of solidarity for their own sake. The principle “it could happen to us” is particularly relevant in the case of the pandemic but could equally apply to all types of crises that contain a threat of contamination. For example, regarding the euro crisis, as Schelkle (2017: 188–189) cogently argued, there was “nothing deterministic in the turn of a common shock into a handful of country crises. It was fundamentally underdetermined whether any particular country would get into deeper troubles and, if so, which one”. When one talks about interstate solidarity in response to the pandemic, this immediately implies that the virus is no longer endemic, meaning that the first trench of defense at the local level has already fallen (Aaltola et al., 2021). Solidarity between member states in fighting the pandemic mainly materialized through the dispatch of medical supplies and treatment of patients. For example, Germany, Poland and Romania sent a team of medical experts to treat patients in Italy; Austria, Belgium, Germany and Luxembourg sent specialized medical staff to those countries in need and treated patients from the Netherlands, France and Italy in their intensive care units; Austria, Hungary and the Netherlands sent ventilators to the Czech Republic; France sent more than two million masks to Italy and to other member states in need; Germany sent five tons of medical supplies, such as ventilators and protective gear (masks and gloves) to Italy (European Council, 2021). Solidarity between member states was, however, not without its challenges: the Czech Republic, Cyprus, Denmark, Hungary, Latvia, Lithuania, Poland, Slovakia and Spain closed their borders to foreign nationals because of fear of contamination, while France and Germany stockpiled personal protective equipment (Schelkle, 2021). In another incident that attracted international media attention, the Czech authorities impounded face masks that were sent as a gift from the Red Cross branch in China to the Chinese community in Italy. The incident was a matter of misunderstanding and was resolved with Czech officials sending around 102,000 confiscated masks back to Italy. Despite these hindrances, a survey commissioned by EUI-YouGov showed that EU citizens were on average supportive of the argument that national governments should allocate national resources, not only in their own but also in other EU countries, with support being stronger among citizens of south/eastern than of northern/western member states (Genschel et al., 2021).

### Data and hypotheses

Data primarily draws on a special Eurobarometer survey (Eurobarometer 95.1)^2^ that was conducted for the European Parliament in 27 countries during the pandemic (March-April 2021). We also employ the Oxford's COVID-19 government response tracker (OxCGRT) to extract information on the “stringency index” and the ‘debt relief’ variable that we use as controls in our regression models (see the Online appendix for a detailed description of the variables).

The EU handles crises through a complex mechanism of decision-making that often delays decisions and weakens the intensity of actions in the pursuit of a broader consensus. A lesson learned from the euro crisis was that the existent interdependence between EU countries made every country in the Union vulnerable to international crisis, regardless of how well prepared they were domestically to absorb the shocks thereof. The late and indecisive reaction of the EU during the euro crisis had severe repercussions as the crisis spread from Greece to other member states such as Cyprus, Spain, Portugal and Ireland, with political consequences also being felt in countries such as France and Italy. In the case of the pandemic, some argue, that despite the initial vacillation regarding the appropriateness or not of certain vaccines against the pandemic, the EU acted decisively promoting solidarity between member states in fighting the pandemic. Still, others are more skeptical and argued not only that the EU measures came late but that competition between member states for medical equipment and supply chain disruptions hampered the ability of regions to respond effectively to the pandemic (Kovács and Sigala, 2021). Indeed, according to Eurobarometer data, the European public is divided on the matter, with 51% expressing dissatisfaction and 49% satisfaction with solidarity between member states in fighting the pandemic.

H1 Citizens’ satisfaction with solidarity between member states in fighting the COVID-19 pandemic positively affects the image of the EU.

Along with satisfaction with interstate solidarity, the Eurobarometer asks citizens to indicate if they are satisfied with the measures taken by the EU in fighting the pandemic (institutional solidarity). If the two concepts of institutional and interstate solidarity were complementary, they would produce sizeable effects in the same direction. This means that the combined effect would increase the positive opinions held by citizens towards the EU. However, we assume a more complex relationship: although interstate and institutional solidarity alone will have a positive effect on EU image, their combination will reduce the additive effect of both. The negative interaction between these two types of solidarity lies in the fact that the EU combines both federal and intergovernmental features, which are deemed as antithetical to one another (Nicolaïdis, 2013). Put differently, the emphasis on either interstate or institutional solidarity echoes the classical distinction between federalists and intergovernmentalists (Nicolaïdis, 2013), meaning that those who believe in the EU as a union of sovereign member states place greater emphasis on the effect of interstate solidarity on the EU image vis-à-vis those advancing the idea of the EU as a federation who are more likely to promote the positive effects of institutional solidarity. Given the above, we expect that citizens who are not satisfied with institutional solidarity will rely more on interstate solidarity and so the effects of the latter on EU image will be more pronounced compared to citizens who expect more from institutional solidarity. In effect, what the EU image gains from solidarity at the interstate level is due to the lacuna of solidarity at the supranational level. Our second hypothesis therefore reads as follows:

H2 The effect of satisfaction with interstate solidarity on EU image will be larger for citizens who are less satisfied with institutional solidarity in fighting the pandemic.

Although all EU member states experienced the detriments of the pandemic, some were hit harder than others. The Czech Republic reported the largest number of deaths from the pandemic relevant to its population^3^ during the period of the Eurobarometer survey^4^. Following the previous discussion of the EU as a union of many *Demoi* with different needs that should be respected (Nicolaïdis, 2013), we expect that the effect of satisfaction with interstate solidarity on EU image will have different sized effects depending on the degree to which the citizens of a country have suffered from the pandemic. A country worst affected by the pandemic is also expected to mobilize other member states to express solidarity. This argument resonates with the growing literature on deservingness, according to which people rely on a deservingness heuristic to decide whether or not recipients are worth of solidarity (Petersen, 2012). If the recipient country is perceived as deserving of help, citizens of other member states support solidarity; if the recipient country is perceived as undeserving, solidarity is not supported. Subsequently, if the recipient country is satisfied with solidarity offered by other member states, this will also have a positive impact on the image of the EU as a whole. So, if our main hypothesis that satisfaction with solidarity has a positive effect on the EU's image is correct, then we expect this effect to be more sizeable in the case of Czech Republic, which is the country that was the most severely affected by the COVID-19 pandemic during the period of the survey.

H3 The effect of satisfaction with solidarity between member states in fighting the COVID-19 pandemic on EU image will be more sizeable in the case of Czech Republic in comparison to all other member states.

## Descriptive statistics

**Figure 1.**
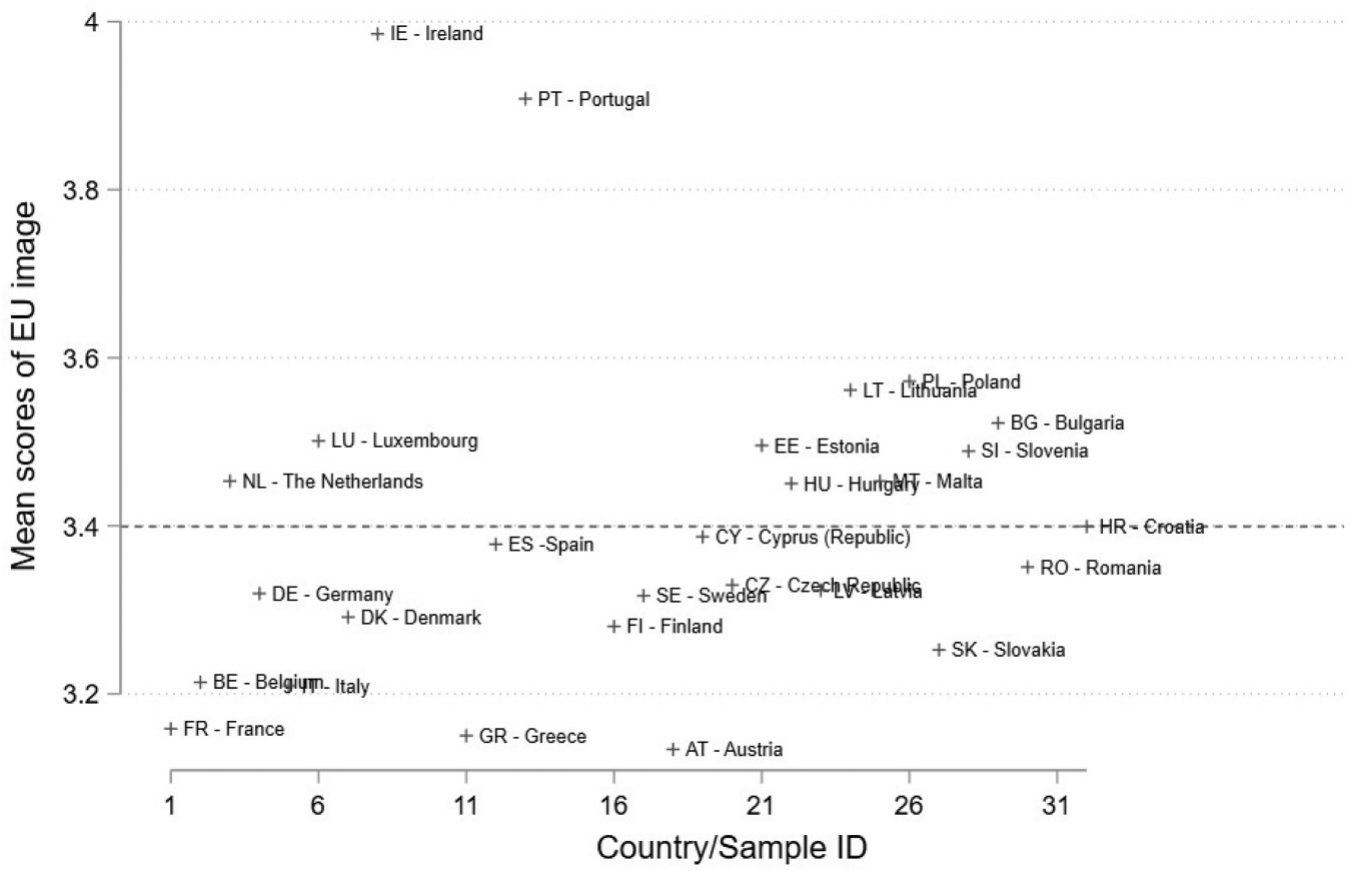
Mean scores of EU image by member state.

Figure 1 presents average scores of EU image stratified by member state. Observations below (above) the dashed horizontal line indicate that the country's mean score is below (above) the EU average (3.4). The citizens of Austria, Greece and France hold on average the least positive views of EU whereas those of Ireland and Portugal the most positive ones. In general member states’ average views on EU image are not very different from one another (N = 27; SD = 0.2).

Satisfaction with interstate solidarity follows a similar distribution in most member states with satisfaction with EU measures, a few exceptions withstanding (the Online appendix also presents the distribution of satisfaction with interstate solidarity and satisfaction with EU measures). The most notable exception involves the Netherlands, and to a lesser degree Sweden, Austria, Latvia, Estonia and Finland. Although one cannot cross out the presence of endogeneity between institutional and interstate solidarity, there is no collinearity problem when both are inserted in the same regression model, as the Pearson correlation equals 0.5. In Denmark, Ireland, Portugal, Poland, Cyprus, Czech Republic, Hungary, Lithuania, Malta, Slovakia, Bulgaria, Romania and Croatia, most citizens of these countries are more satisfied with interstate solidarity in fighting the COVID-19 pandemic than dissatisfied. Further, in the Netherlands, Denmark, Portugal, Finland, Sweden, Austria, Cyprus, Czech, Estonia, Hungary, Latvia, Lithuania, Malta, Poland, Slovakia, Bulgaria, Romania and Croatia, most citizens are more satisfied with EU measures in fighting the COVID-19 pandemic than dissatisfied.

**Figure 2.**
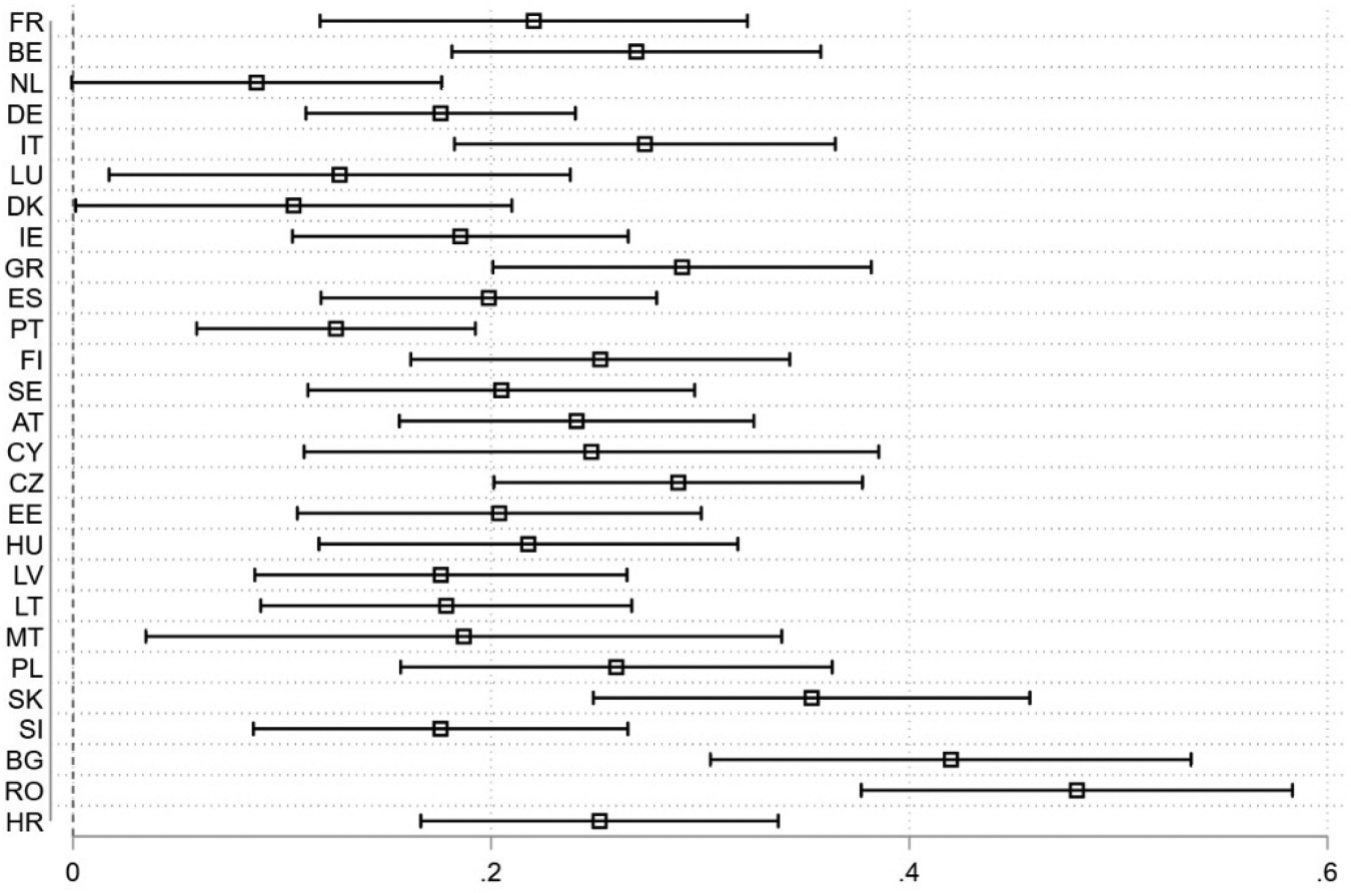
Effect of satisfaction with interstate solidarity on EU image by member state.

Notes: The analysis controls for ‘Corona virus impact on personal income’; ‘Debt relief dummy (OxCGRT)’; ‘Stringency index (OxCGRT)’; ‘Left and right ideology’; ‘Education’; ‘Employment status’; ‘Social class’; ‘Gender’; ‘Marital status’; ‘Age’ and ‘Age-square’; See Figure 1 for the countries’ abbreviations.

Figure 2 shows the coefficient estimates from linear regressions for each of the 27 countries separately, where the EU's image is the dependent variable and satisfaction with interstate solidarity the main independent one. The analysis also controls for the effect of satisfaction with EU measures as well as other independent variables^5^ (see notes in Figure 2), but their coefficient estimates have been supressed from showing for better clarity. In all cases save one, results show that satisfaction with interstate solidarity has a positive effect on the EU's image, meaning that as satisfaction with interstate solidarity increases, citizens’ views on the EU also become more positive. The only exception is that of the Netherlands: although the estimated coefficient of satisfaction with interstate solidarity is towards the anticipated order (positive) it is nevertheless associated with a probability value just above the alpha level (p = .052, alpha = .05, N = 736).

## Model specification and findings

Considering the hierarchical nature of our data, our model specification is a linear mixed model where the intercept is allowed to vary by twenty-seven countries and standard errors are clustered at the level of member states. Although, a simple model where interstate solidarity is the only independent variable has an intraclass correlation coefficient (ICC) close to zero (ICC = .04, SE = .015), a likelihood ratio (LR) test clearly indicates that a mixed model should be preferred over a flat linear regression (LR χ²(2) = -32853.93, p < .001).

Model 1 (Table 1) tests for *H1* assuming that the EU's image is only affected by satisfaction with interstate solidarity. Results show that satisfaction with interstate solidarity has a positive effect on the EU's image. Model 2 (Table 1) adds a series of controls^6^ on both the individual and country level. Before discussing the effect that our main independent variable has on the outcome, we first present the effect of control variables. Results show that those whose income has not been or will not be affected by COVID-19 have a more positive image of EU than those whose personal income has already been impacted by the pandemic. In member states where the government halted financial obligations for households, the EU has a less positive image than in member states in which the government did not offer debt relief. We believe this is because households relied more on EU financial assistance to tackle the negative financial consequences caused by the COVID-19 pandemic in member states that did not offer a debt relief. On the other hand, the strictness of lockdown does not seem to impact citizens’ image of the EU. Similarly, left and right ideology, employment, and marital status do not seem to affect citizens’ views on the EU in either positive or negative ways. On the other hand, those with a university education are more likely to have a more positive view on the EU compared to those with only a primary education. Additionally, those belonging to middle, upper middle, and higher class have more positive views of the EU compared to those belonging to the working class. Males have more positive views on the EU than females but the effect is rather small. Lastly, age appears to have a curvilinear effect on the outcome: the EU's image declines in younger ages, but as people become older, they start having more positive views of the EU. Regarding our main independent variable, it should be noticed that after controlling for the effect of all these variables, satisfaction with interstate solidarity not only keeps its statistical significance (with p < .001) but also the size of the effect remains almost unchanged (compared with coefficient estimate in Model 1). In Model 3 (Table 1), we add one more regressor, satisfaction with EU measures against the COVID-19 pandemic (i.e., satisfaction with institutional solidarity). We first notice that all other control variables behave more or less as explained in Model 2 (Table 1). The coefficient estimate of satisfaction with institutional solidarity is, as in the case of interstate solidarity, positive and statistically significant, indicating that as satisfaction with institutional solidarity increases, the EU's image becomes more positive. When adding satisfaction with institutional solidarity in the list of regressors, the coefficient estimate of satisfaction with interstate solidarity drops from 0.43 (Model 2) to 0.23 (Model 3), yet it retains its statistical significance.

**Table 1.** Linear mixed models.

	Model 1	Model 2	Model 3	Model 4	Model 5
Interstate solidarity	.444*** (.021)	.429*** (.021)	.234*** (.016)	.465*** (.031)	.226*** (.018)
Institutional solidarity			.411*** (.025)	.633*** (.038)	.411*** (.026)
Interstate solidarity × Institutional solidarity				-.094*** (.011)	
Czech Republic (D)					-.654*** (.092)
Interstate solidarity × Czech Republic (D)					.184*** (.019)
Impact on income: Coronavirus has not yet impacted on my income Coronavirus will have no impact on my income		.036* (.018) .098*** (.017)	.004 (.017) .061*** (.016)	.001 (.018) .060*** (.016)	.005 (.017) .060*** (.016)
Debt relief (D) (OxCGRT)		-.212*** (.008)	-.197*** (.007)	-.204*** (.006)	.047 (.077)
Stringency index (OxCGRT)		.007 (.005)	.007 (.005)	.007 (.005)	.006 (.003)
Ideology		-.016 (.013)	-.014 (.011)	-.012 (.011)	-.014 (.011)
Education: Secondary Postsecondary Tertiary		.059 (.048) .044 (.056) .247*** (.049)	.013 (.048) -.010 (.060) .173** (.052)	.013 (.048) -.010 (.060) .170** (.052)	.015 (.048) -.009 (.061) .175** (.052)
Employment status: Unemployed		-.028 (.016)	-.008 (.013)	-.009 (.014)	-.010 (.014)
Social class: Lower middle Middle class Upper middle class Higher class		.045 (.036) .143*** (.035) .301*** (.038) .302*** (.073)	.061 (.033) .145*** (.034) .286*** (.039) .302*** (.074)	.059 (.033) .143*** (.034) .283*** (.038) .303*** (.074)	.062 (.033) .144*** (.034) .284*** (.039) .305*** (.073)
Gender: Male		.036* (.017)	.053** (.018)	.055** (.018)	.053** (.018)
Marital status: Married		.013 (.013)	.016 (.015)	.016 (.015)	.017 (.015)
Age		-.018*** (.004)	-.014*** (.004)	-.014** (.004)	-.014*** (.004)
Age²		.000*** (.000)	.000** (.000)	.000** (.000)	.000** (.000)
Country dummies	No	Yes	Yes	Yes	No†
Random-effects parameters: Variance of the level-two errors Variance of the level-one errors	.029 (.011) .717 (.027)	2.04e-13 (1.07e-08) .691 (.026)	2.42e-13 (7.66e-13) .607 (.020)	2.40e-13 (7.39e-13) .603 (.021)	.032 (.009) .607 (.020)
N	26,192	24,090	20,505	20,505	20,505

Notes: Reference categories: ‘Coronavirus has already impacted on my personal income’ (Impact on income); ‘The government did not halt financial obligations for households’ (Debt relief (D)); Primary (Education); ‘Self-employed/employed’ (Employment status); ‘Working class’ (Social class); ‘Citizen of any other member’ state’ (Czech Republic (D)); ‘‘Female’ (Gender); ‘Living alone (single/divorce/widow) (Marital status); ***p < .001, **p < .01, *p < .05; alpha = 0.5; parenthesized entries are errors clustered at the country level. † The reason for not including country dummies in this regression model is due to collinearity with the dummy for Czech Republic (1: if citizen of Czech Republic; 0: If citizen of any other member state); See the Online appendix for a detailed description of the variables.

Turning now our attention to Model 4 (Table 1), we see that the interaction term is negative and statistically significant. This means that the effect of interstate solidarity on the EU's image weakens as one becomes more satisfied with EU measures against the pandemic. Table 2 presents the results from comparing the slope coefficients of EU image on interstate solidarity for different levels of satisfaction with EU measures against the pandemic.

**Table 2.** Average marginal effects on satisfaction with interstate solidarity.

Pair comparisons	AMEs on satisfaction with interstate solidarity	Difference in AMEs
Not at all satisfied with EU measures Rather not satisfied with EU measures	.371*** [.328 .413] .277*** [.246 .307]	-.09***
Not at all satisfied with EU measures Rather satisfied with EU measures	.371*** [.328 .413] .183*** [.15 .216]	-.19***
Not at all satisfied with EU measures Very satisfied with EU measures	.371*** [.328 .413] .089*** [.042 .14]	-.28***
Rather not satisfied with EU measures Rather satisfied with EU measures	.277*** [.246 .307] .183*** [.15 .216]	-.09***
Rather not satisfied with EU measures Very satisfied with EU measures	.277*** [.246 .307] .089*** [.042 .14]	-.19***
Rather satisfied with EU measures Very satisfied with EU measures	.183*** [.15 .216] .089*** [.042 .14]	-.09***

Notes: χ²(1) = 67.38; ***p < .001; CI 95% inside brackets.

The largest difference in average marginal effects (AMEs) is observed when we compare the effect that satisfaction with interstate solidarity has on the EU's image for those respondents who are not at all satisfied with EU measures against those who are very satisfied. We see that the variable concerning the EU's image changes on average by 0.371 units for every unit change in interstate solidarity when respondents are not at all satisfied with EU measures. On the other hand, EU image changes on average by a mere 0.089 units for a unit change of satisfaction with interstate solidarity for those respondents who are very satisfied with EU measures. The second largest difference in AMEs is observed when we compare the slope coefficients of EU image on satisfaction with interstate solidarity between the pairs: “Not at all satisfied with EU measures” vs. “Rather satisfied with EU measures” and “Very satisfied with EU measures” vs. “Rather not satisfied with EU measures” (all other differences in AMEs are smaller in size).

**Figure 3.**
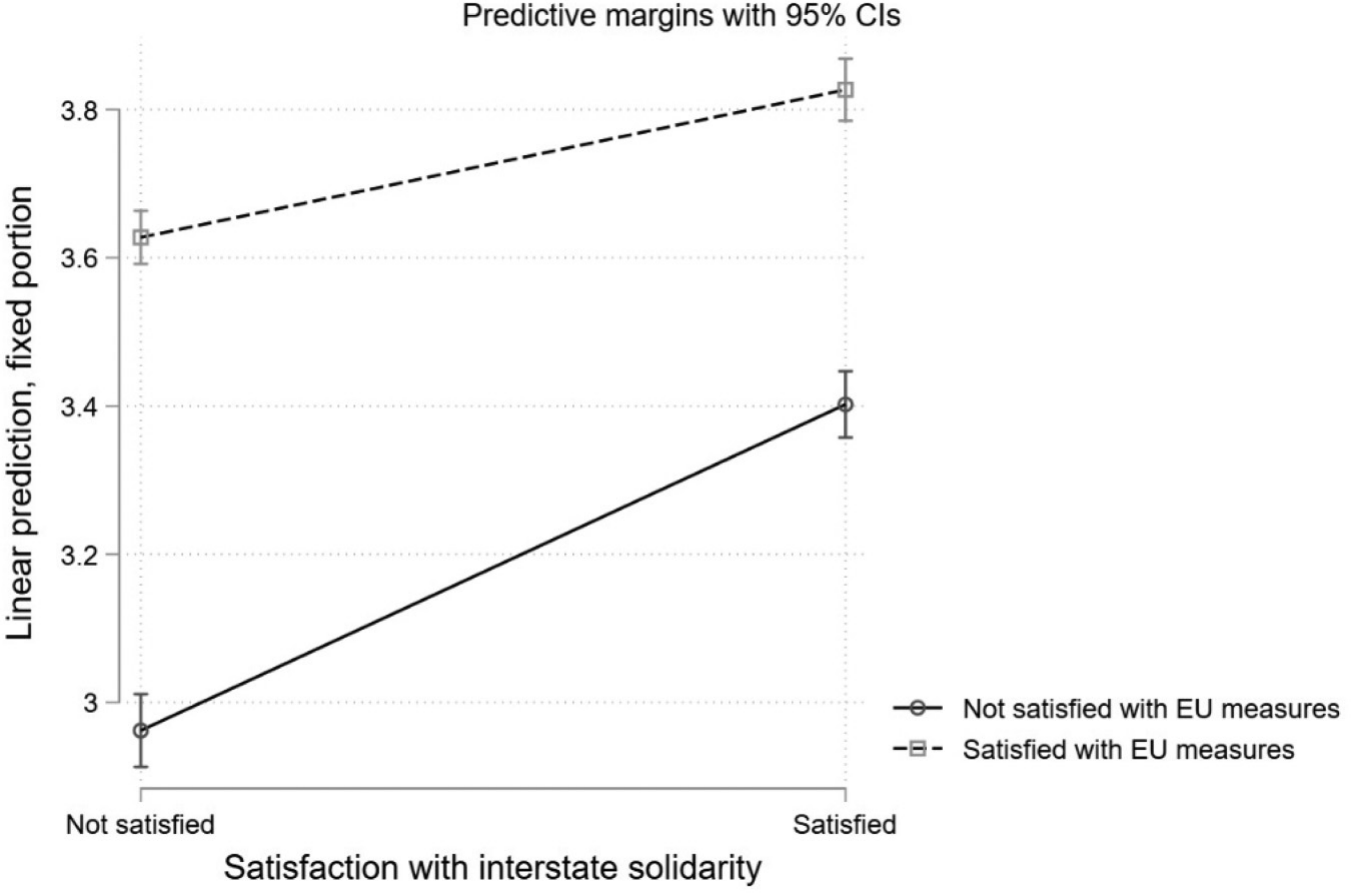
Effect of interstate solidarity on EU image for different levels of institutional solidarity.

Without loss of generality, we now treat both satisfaction with interstate solidarity and satisfaction with EU measures in fighting the COVID-19 pandemic as dummies and estimate the coefficient of the interaction in the regression. Figure 3 shows the effect of satisfaction with interstate solidarity (0: Not satisfied with interstate interaction; 1: Satisfied with interstate interaction) on EU image for respondents who are satisfied with EU measures (1) and those who are not (0). Once more we see that those respondents who are not satisfied with EU measures hold, on average, a less positive view on the EU compared to those who are satisfied with EU measures against the COVID-19 pandemic. The findings confirm the *H2*, as the gradient of the line that shows the relationship between satisfaction with interstate solidarity and EU image is less steep in the case of those who are satisfied with EU measures than those who are not (Figure 3). On average the effect of satisfaction with interstate solidarity on EU image is .24 units smaller (p < .001) for respondents who are satisfied with EU measures compared to respondents who are not satisfied with EU measures.

**Figure 4.**
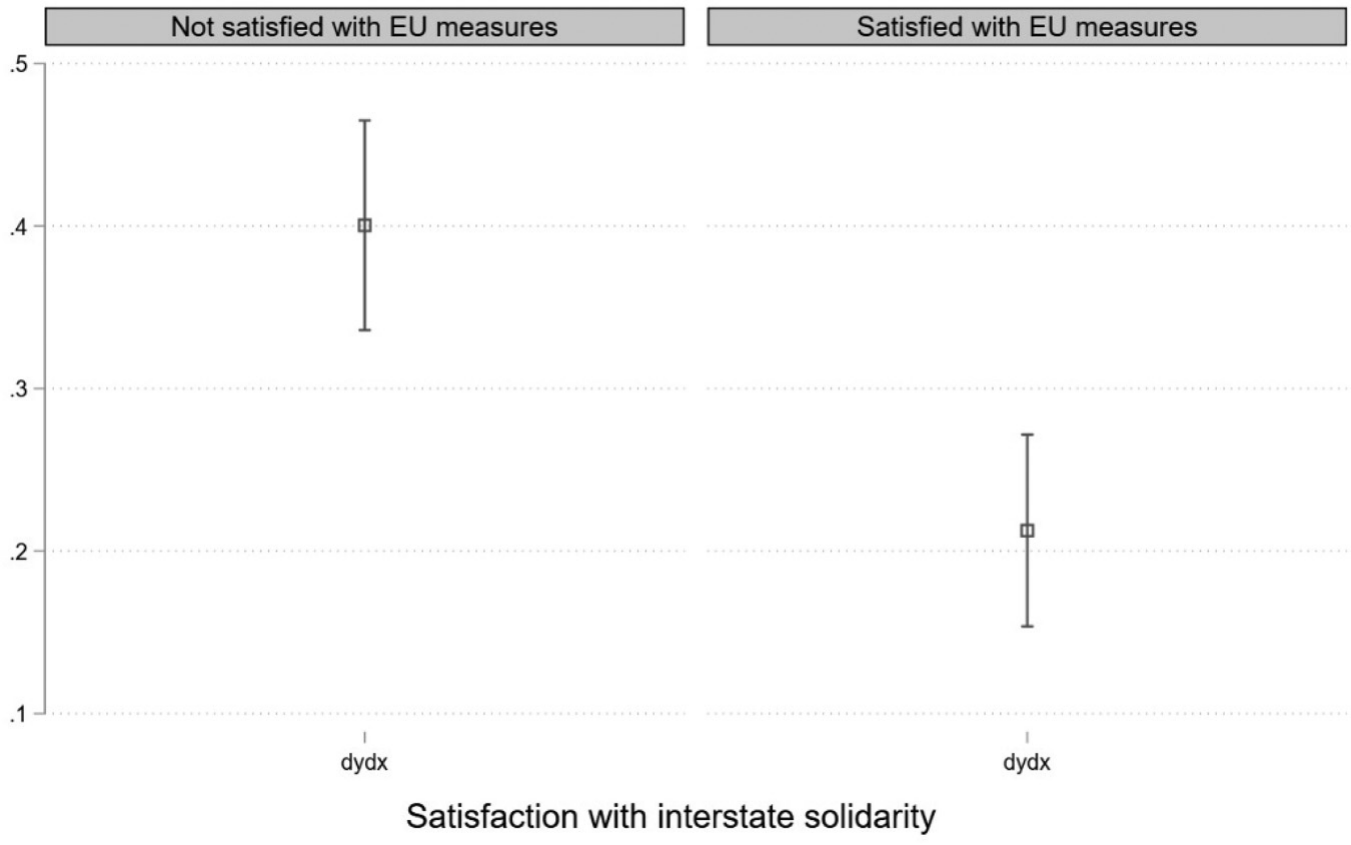
Effect of interstate solidarity on EU image for exact matched samples.

Following Bhatti et al. (2020), we also test this finding by following a different methodology. First, we split the sample into two groups: (a) Respondents who are not satisfied with EU measures in fighting the COVID-19 pandemic (44.62% of the total sample); (b) Respondents who are satisfied with EU measures in fighting the pandemic (55.38% of the total sample) (N = 22,084). Employing then the Coarsened Exact Matching algorithm (Iacus et al., 2009) and using as treatment dichotomous variable interstate solidarity (0: Not satisfied with interstate solidarity, 1: Satisfied with interstate solidarity), we match the two groups on the following pre-treatment characteristics: debt relief (two categories), stringency index (we coarsen the variable into bins for every two units spanning from 43 to 89), coronavirus impact on personal income (three categories), left and right (ten categories), marital status (two categories), class (five categories), education (four categories), gender (two categories), employment status (two categories) and age (we coarsen the variable into bins for every three years spanning from 18 to 90). Because we employ exact matching, our analysis contains the same number of treated and control units for each group—in the group “Not satisfied with EU measures“, we do not match 5112 (4679) respondents who are not satisfied with interstate solidarity (are satisfied with interstate solidarity); in the group “Satisfied with EU measures”, we do not match 6112 (5679) respondents who are not satisfied with interstate solidarity (are satisfied with interstate solidarity). Finally, our analysis matches 8214 observations for the group “Not satisfied with EU measures” and 7214 observations for the group “Satisfied with EU measures”. In both cases, our design contains an adequate number of observations for a regression analysis. Lastly, considering only the matched cases we run two different multilevel linear models, one for each group. As expected, we find that the effect of interstate solidarity on EU image is larger for the group of respondents who are not satisfied with EU measures compared to those who are satisfied with no overlapping confidence intervals between the two coefficient estimates (Figure 4).

This finding is important as it shows that the combined effect of satisfaction with interstate and institutional solidarity is more complex than the main effect that each of these has on EU image individually. Although both interstate and institutional solidarity have a positive effect on EU image (Model 3, Table 2), when considering the interaction of the two, we see that citizens have the most positive image of the EU when they are not satisfied with institutional solidarity while at the same time satisfied with interstate solidarity. A possible explanation for this finding is that citizens who are not satisfied with EU measures in fighting the pandemic (low level of institutional solidarity) have a less positive image of EU and therefore there is more *space* left for satisfaction with interstate solidarity to *fill in*.

**Figure 5.**
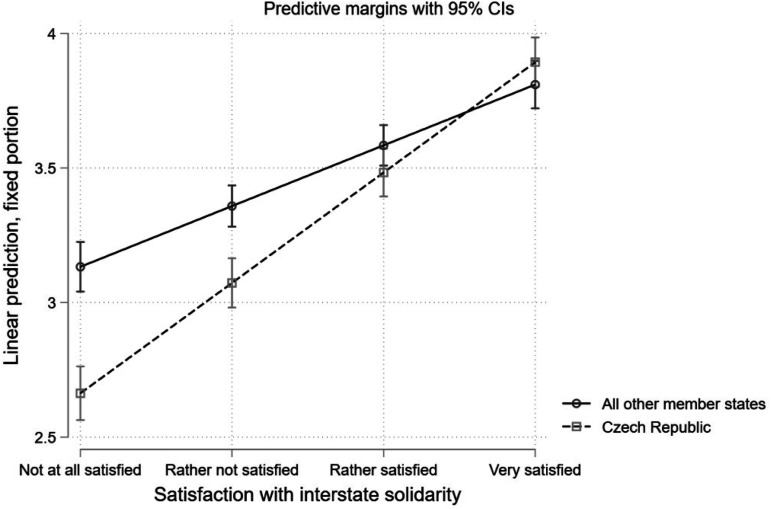
Effect of satisfaction with interstate solidarity on EU image for Czech citizens.

The last model (Model 5, Table 2) tests *H3* by the means of an interaction between satisfaction with interstate solidarity and a dummy variable that captures if the respondent is a citizen of Czech Republic (1) or not (0). All control variables behave the same as in the case of Model 3 (Table 2), apart from the coefficient estimate of the debt relief dummy that is not statistically significant. The coefficient estimate of the interaction is positive and statistically significant and the slope of satisfaction with interstate solidarity in the case of Czech Republic is .18 units steeper compared to the slope of all other member states. Figure 5 plots the predictive margins separately for Czech Republic and all other member states for different values of satisfaction with interstate solidarity. Results testify that the difference in predictive margins between Czech Republic and all other member states, which is the largest for citizens not at all satisfied with interstate solidarity, becomes smaller as satisfaction with interstate solidarity increases. In general, we see that Czech citizens have a less positive image of the EU compared to all other EU citizens for different values of satisfaction with interstate solidarity—which suggests that perceived absence of solidarity matters more for EU image than its perceived presence (only those Czech citizens who are very satisfied with interstate solidarity seem to have a more positive image of the EU compared to all other EU citizens).

## Conclusions

Although the COVID-19 pandemic was not the first but the third large crisis—after the euro and the so-called refugee crises—during the last decade in Europe, it was however the first crisis where all EU countries felt the Sword of Damocles hanging above their heads. The EU responded to the COVID-19 pandemic by promoting both institutional and interstate solidarity.

Employing data from a special Eurobarometer survey enriched with data from OxCGRT, we can derive the following three conclusions: first, EU citizens who were more satisfied with solidarity between member states in fighting the COVID-19 pandemic had a more positive image of the EU compared to citizens who were less satisfied. Second, satisfaction with interstate solidarity had a more sizeable effect on the EU image for those citizens who were less satisfied with institutional solidarity (i.e., less satisfied with EU measures against the pandemic) than more satisfied. Third, the effect of satisfaction with interstate solidarity on the EU image variable was more sizeable in the case of Czechs, whose country was hit hard by the pandemic. These findings highlight how in times of crisis actions of solidarity between members of the same institution can have a positive effect on the image of the organization itself, even if its actions in handling the crisis are deemed unsatisfactory.

Overall, our results demonstrate that the pandemic has done for the EU what the Lisbon Treaty's Solidarity clause only aspired to. The global and overarching nature of the crisis has shown EU citizens that cooperation, mutual aid and solidarity were clearly required to combat it. Furthermore, EU citizens are not significantly interested in whether it is the EU itself that coordinates the actions of solidarity or only its member states as long something is achieved. Ultimately, all of the solidarity measures in the eyes of the citizens improve their view of the EU as a whole, which ought to show that the EU should further encourage solidarity leading by example or coaxing members to act on their own accord.

A caveat of our analysis is that the Eurobarometer survey was conducted in a period in which vaccines against the COVID-19 pandemic had already become available and therefore the image of the EU was more positive compared to the onset of the pandemic, a period characterized by uncertainty and fear. However, we believe that if interstate solidarity can have a positive impact on the EU in a period where its image is generally positive, the effect might even be more pronounced in a period where its image is negative. Another caveat is that although we study the interaction between important types of solidarity, such as interstate and institutional solidarity, data restrictions do not permit us to expand our analysis to interpersonal solidarity between European citizens, which is another relevant form of solidarity (Lahusen and Theiss, 2019). Lastly, although it would be concerned with assessing how the effect of satisfaction with solidarity might fluctuate depending on different levels of “Europeanness”, a variable that captures citizens’ degree of attachment to European identity was not included in this special Eurobarometer data.

Our article is the first to research the effect of interstate solidarity on the image of the EU in a period of crisis, the COVID-19 pandemic, and paves the way for further research on the topic. For example, future research could aspire to compare the effect that satisfaction with interstate solidarity has on the EU's image between different types of crises, e.g., a global pandemic versus a public debt crisis. Although such analysis might have to deal with the caveats of comparing findings from different periods of time, it would nevertheless be interesting to see if satisfaction with interstate solidarity would impact the views of the EU's image for types of crises that are likely to trigger different reactions of solidarity (Katsanidou et al., 2022).

## Supplemental Material

sj-pdf-1-eup-10.1177_14651165231156846 - Supplemental material for How supranational institutions benefit from crises: Member states’ solidarity and the EU's image during the COVID-19 pandemic Click here for additional data file. Supplemental material, sj-pdf-1-eup-10.1177_14651165231156846 for How supranational institutions benefit from crises: Member states’ solidarity and the EU's image during the COVID-19 pandemic by Achillefs Papageorgiou and Waltteri Immonen in European Union Politics

sj-do-2-eup-10.1177_14651165231156846 - Supplemental material for How supranational institutions benefit from crises: Member states’ solidarity and the EU's image during the COVID-19 pandemic Click here for additional data file. Supplemental material, sj-do-2-eup-10.1177_14651165231156846 for How supranational institutions benefit from crises: Member states’ solidarity and the EU's image during the COVID-19 pandemic by Achillefs Papageorgiou and Waltteri Immonen in European Union Politics

sj-dta-3-eup-10.1177_14651165231156846 - Supplemental material for How supranational institutions benefit from crises: Member states’ solidarity and the EU's image during the COVID-19 pandemic Click here for additional data file. Supplemental material, sj-dta-3-eup-10.1177_14651165231156846 for How supranational institutions benefit from crises: Member states’ solidarity and the EU's image during the COVID-19 pandemic by Achillefs Papageorgiou and Waltteri Immonen in European Union Politics

## Notes

1. This special Eurobarometer survey includes a battery of questions regarding the NGEU package, therefore the term “measures” mostly refers to this (see the paragraph above).

2. Data available at https://search.gesis.org/research_data/ZA7781?doi=10.4232/1.13791 and https://data.europa.eu/data/datasets/s2572_95_1_eng?locale=en (accessed 10 January 2023).

3. Data available at https://github.com/owid/covid-19-data/tree/master/public/data (accessed 10 January 2023)—we thank an anonymous reviewer for pointing this out. Also, note that the Czech Republic also leads the table of “Cumulative confirmed COVID-19 cases per million people”.

4. If one considers number of deaths irrespectively of the country's population, Italy is the country that was the most severely affected by the COVID-19 pandemic during the period of the survey. The Online appendix presents results of Model 5 assuming an interaction between satisfaction with interstate solidarity and a dummy variable that captures if the respondent is a citizen of Italy (1) or not (0). Results point to the same findings.

5. Similarly to relevant recent publications on solidarity (e.g. Katsanidou et al., 2022; Haverland et al., 2022), we also control for idiosyncratic variables such as gender, education, marital status, etc.

6. See the Online appendix for an alternative analysis. Results show that findings are robust to variables’ selection.

## References

[bibr1-14651165231156846] Aaltola M Ketola J Vaakanainen K, et al. (2021) Solidarity during COVID-19 at national regional and global levels. Finnish Institute for International Affairs. Briefing paper. May 2021/309.

[bibr2-14651165231156846] Armingeon K de la Porte C Heins E, et al. (2022) Voices from the past: Economic and political vulnerabilities in the making of next generation EU. Comparative European Politics 20: 144–165.

[bibr3-14651165231156846] Banting K Kymlicka W (2017) The Strains of Commitment: The Political Sources of Solidarity in Diverse Societies. New York: Oxford University Press.

[bibr4-14651165231156846] Bayertz K (1999) Four uses of “solidarity”. In: Bayertz K (ed) Solidarity. Dordrecht: Springer, pp. 3–28.

[bibr5-14651165231156846] Bhatti A Akram H Basit HM, et al. (2020) E-commerce trends during COVID-19 pandemic. International Journal of Future Generation Communication and Networking 13(2): 1449–1452.

[bibr6-14651165231156846] Christie R Claeys G Weil P (2021) Next Generation EU borrowing: a first assessment. *Policy Contribution* 22/2021, Bruegel.

[bibr7-14651165231156846] Cicchiello AF Cotugno M Monferrà S, et al. (2022) Credit spreads in the European green bond market: A daily analysis of the COVID-19 pandemic impact. Journal of International Financial Management & Accounting 33(3): 1–29.

[bibr8-14651165231156846] European Commission (2020) Coronavirus: European Solidarity in action. Available at: https://ec.europa.eu/info/live-work-travel-eu/coronavirus-response/coronavirus-european-solidarity-action_en (accessed 10 January 2023).

[bibr9-14651165231156846] European Commission (2021) Recovery and Resilience Facility. Available at: https://ec.europa.eu/info/business-economy-euro/recovery-coronavirus/recovery-and-resilience-facility_en#national-recovery-and-resilience-plans (accessed 10 January 2023).

[bibr10-14651165231156846] European Council (2021) European solidarity in action. Available at: https://www.consilium.europa.eu/en/policies/coronavirus/european-solidarity-in-action/ accessed 10 January 2023).

[bibr11-14651165231156846] European Investment Bank (2022) The European Investment Bank and COVID-19. Available at: https://www.eib.org/en/about/initiatives/covid-19-response/index.htm (accessed 10 January 2023).

[bibr12-14651165231156846] Ferrera M Burelli C (2019) Cross-national solidarity and political sustainability in the EU after the crisis. Journal of Common Market Studies 57(1): 94–110.

[bibr13-14651165231156846] Genschel P Jachtenfuchs M (2021) Postfunctionalism reversed: Solidarity and rebordering during the COVID-19 pandemic. Journal of European Public Policy 28(3): 350–369.

[bibr14-14651165231156846] Genschel P Hemerijck A Nasr M, et al. (2021) Solidarity and trust in the times of COVID-19. RSC Policy Paper 2021/11. European University Institute. Available at: https://cadmus.eui.eu/handle/1814/73131 (accessed 10 January 2023).

[bibr15-14651165231156846] Goldberg AC Gattermann K Marquart F, et al. (2021) European Solidarity in times of crisis: The role of information and media use. West European Politics 44(5-6): 1314–1328.

[bibr16-14651165231156846] Haverland M van der Veer R Onderco M (2022) Is this crisis different? Attitudes towards EU fiscal transfers in the wake of the COVID-19 pandemic. European Union Politics. (forthcoming). 10.1177/14651165221112988 PMC9277315 38603186

[bibr17-14651165231156846] Howarth D Kavvadia H Clifton J, et al. (2020) The role of the European investment bank in times of COVID-19. In: McDonald DA Marois T Barrowclough DV (eds) Public Banks and COVID-19: Combatting the Pandemic with Public Finance. Publisher: UNCTAD, pp. 135–148.

[bibr18-14651165231156846] Iacus SM King G Porro G (2009) Cem: Software for coarsened exact matching. Journal of Statistical Software 30(9): 1–27.21666874

[bibr19-14651165231156846] Jones E (2021) Next Generation EU: Solidarity, opportunity, and confidence. European Policy Analysis June 2021:11. Swedish Institute for European Policy Analysis. Available at: https://www.sieps.se/en/publications/2021/next-generation-eu-solidarity-opportunity-and-confidence/ (accessed 10 January 2023).

[bibr20-14651165231156846] Katsanidou A Otjes S (2016) How the European debt crisis reshaped national political space: The case of Greece. European Union Politics 17(2): 262–284.

[bibr21-14651165231156846] Katsanidou A Reinl AK Eder C (2022) Together we stand? Transnational solidarity in the EU in times of crises. European Union Politics 23(1): 66–78.

[bibr22-14651165231156846] Khushf G (1999) Solidarity as a moral and political concept: Beyond the liberal/communitarian impasse. In: Bayertz K (ed) Solidarity. Dordrecht: Springer, pp. 57–79.

[bibr23-14651165231156846] Klamert M (2022) Loyalty and solidarity as general principles. In: Ziegler KS Neuvonen PJ Moreno-Lax V (eds) Research Handbook on General Principles in EU Law. Cheltenham: Edward Elgar, pp. 118–135.

[bibr24-14651165231156846] Kovács G Sigala IF (2021) Lessons learned from humanitarian logistics to manage supply chain disruptions. J Supply Chain Manag 57: 41–49.

[bibr25-14651165231156846] Lahusen C Theiss M (2019) European Transnational solidarity: Citizenship in action? American Behavioral Scientist 63(4): 444–458.

[bibr26-14651165231156846] Lindner V (2022) Solidarity without conditionality: Comparing the EU Covid-19 safety nets SURE, pandemic Crisis Support, and European Guarantee Fund SAFE Working Paper, No. 333, Leibniz Institute for Financial Research SAFE, Frankfurt a. M., 10.2139/ssrn.4000612.

[bibr27-14651165231156846] Nicolaïdis K (2003) Our European demoicracy: Is this constitution a third way for Europe?’. In: Nicolaïdis K Weatherill S (eds) Whose Europe? National Models and the Constitution of the European Union. Oxford: Oxford University Print, pp. 137–152.

[bibr28-14651165231156846] Nicolaïdis K (2012) The idea of European demoicracy. In: Dickson J Eleftheriadis P (eds) Philosophical Foundations of European Union Law. Oxford: Oxford University Press, pp. 247–274.

[bibr29-14651165231156846] Nicolaïdis K (2013) European Demoicracy and its crisis. Journal of Common Market Studies 51(2): 351–369.

[bibr31-14651165231156846] Petersen MB (2012) Social welfare as small-scale help: Evolutionary psychology and the deservingness heuristic. American Journal of Political Science 56(1): 1–16. 22375300 10.1111/j.1540-5907.2011.00545.x

[bibr32-14651165231156846] de la Porte C Jensen MD (2021) The next generation EU: An analysis of the dimensions of conflict behind the deal. Social Policy and Administration 55(2): 1–402.

[bibr33-14651165231156846] Parijs PV (2004) Cultural diversity versus economic solidarity: Is there a tension? How must it be Resolved. Bruxelles: De Boeck & Larcier s.a.

[bibr34-14651165231156846] Reinl AK (2022) Transnational solidarity within the EU: Public support for risk-sharing and redistribution. Social Indicators Research 163: 1373–1397. 35669552 10.1007/s11205-022-02937-2 PMC9137446

[bibr35-14651165231156846] Rhodes M (2021) ‘Failing forward’: A critique in light of COVID-19. Journal of European Public Policy 28(10): 1537–1554.

[bibr36-14651165231156846] Schelkle W (2017) The Political Economy of Monetary Solidarity: Understanding the Euro Experiment. Oxford: Oxford University Press.

[bibr37-14651165231156846] Schelkle W (2021) Fiscal integration in an experimental union: How path-breaking was the EU's response to the COVID-19 pandemic? Journal of Common Market Studies 59(S1): 44–55. 34908576 10.1111/jcms.13246 PMC8657515

[bibr38-14651165231156846] Schelkle W (2022) Monetary solidarity in Europe: can divisive institutions become ‘moral opportunities’? Review of Social Economy. doi:10.1080/00346764.2022.2042728. PMC9970183 36864903

[bibr39-14651165231156846] Spielberger L (2022) The politicisation of the European Central Bank and its emergency credit lines outside the Euro Area. Journal of European Public Policy. doi:10.1080/13501763.2022.2037688.

[bibr40-14651165231156846] Tajfel H (1981) Human Groups and Social Categories—Studies in Social Psychology. Cambridge: Cambridge University Press.

[bibr41-14651165231156846] Tesche T (2022) Pandemic politics: The European Union in times of the coronavirus emergency. Journal of Common Market Studies 60(2): 480–496.

[bibr42-14651165231156846] Theodoropoulou S (2022) Recovery, resilience and growth regimes under overlapping EU conditionalities: The case of Greece. Comparative European Politics 20: 201–219.

[bibr43-14651165231156846] Tuomela R (2013) Social Ontology: Collective Intentionality and Group Agents. Oxford: Oxford University Press.

[bibr30-14651165231156846] Unger D Unger-Sirsch J Stockemer D, et al. (2023) What guides citizen support for redistributive EU measures as a response to COVID-19: Justice attitudes, self-interest or support for European integration? European Union Politics. (forthcoming).

[bibr44-14651165231156846] Wilmès S Macron E Mitsotakis K, et al. (2020) [Letter to Charles Michel] March 2020. Available at: https://www.governo.it/sites/new.governo.it/files/letter_michel_20200325_eng.pdf (accessed 10 January 2023).

