# Supplementary material for: How supranational institutions benefit from crises: Member states’ solidarity and the EU's image during the COVID-19 pandemic
Source: Eur Union Polit. 2023 Mar 3:14651165231156846. doi: 10.1177/14651165231156846 (PMC9988624; doi:10.1177/14651165231156846)
Supplement: sj-pdf-1-eup-10.1177_14651165231156846 - Supplemental material for How supranational institutions benefit from crises: Member states’ solidarity and the EU's image during the COVID-19 pandemic [file sj-pdf-1-eup-10.1177_14651165231156846.pdf]

Online appendix

How supranational institutions benefit from crises: member states' solidarity and the EU's image during the COVID-19 pandemic.

Achillefs Papageorgiou and Waltteri Immonen

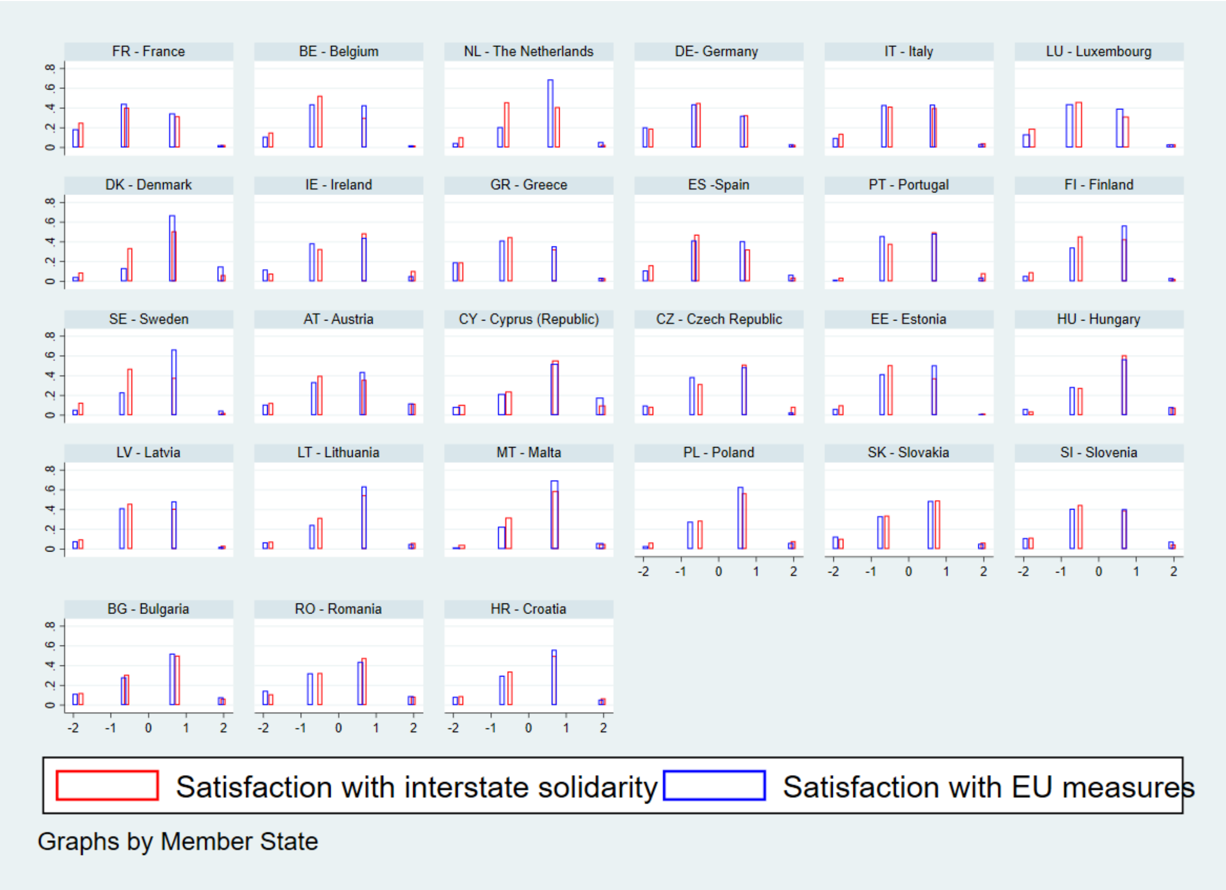

Figure A1. Distribution of satisfaction with interstate solidarity and satisfaction with EU measures.

Table A1. Linear mixed model that includes an interaction between satisfaction with interstate solidarity and a dummy that takes the value of 1 if the respondent is an Italian citizen and 0 otherwise.

	Model 5
Interstate solidarity	.232***
	(.016)
Institutional solidarity	.410***
	(.025)
Italy (D)	-.290**
	(.095)
Interstate solidarity × Italy (D)	.064**
	(.021)
Impact on income:	
Coronavirus has not yet impacted on my income	.004
	(.018)
Coronavirus will have no impact on my income	.060***
	(.017)
Debt relief (D) (OxCGRT)	.044
	(.078)
Stringency index (OxCGRT)	.006
	(.003)
Ideology	-.014
	(.011)
Education:	
Secondary	.013
	(.048)
Postsecondary	-.011
	(.061)
Tertiary	.174**
	(.051)
Employment status: Unemployed	-.008
	(.013)
Social class:	
Lower middle	.061
	(.033)
Middle class	.144***
	(.034)
Upper middle class	.284***
	(.039)
Higher class	.301***
	(.074)
Gender: Male	.053**
	(.018)
Marital status: Married	.017
	(.015)
Age	-.014***
	(.004)
Age²	.000**

	(.000)
Country dummies	No†
Random-effects parameters:	
Variance of the level-two errors	.033
	(.009)
Variance of the level-one errors	.607
	(.021)
N	20,505

Notes: Reference categories: 'Coronavirus has already impacted on my personal income' (Impact on income); 'The government did not halt financial obligations for households' (Debt relief (D)); Primary (Education); 'Self-employed/employed' (Employment status); 'Working class' (Social class); 'Citizen of any other member state' (Italy (D)); ''Female' (Gender); 'Living alone (single/divorce/widow) (Marital status); ***p<.001, **p<.01, *p<.05; alpha=0.5; parenthesized entries are errors clustered at the country level. † The reason for not including country dummies in this regression model is due to collinearity with the dummy for Italy (1: if citizen of Italy; 0: If citizen of any other member state); See Appendix A for a detailed description of the variables.

Table A2. Linear mixed models (Model 2-Model 5) excluding gender and marital status from the list of control variables

	Model 2	Model 3	Model 4	Model 5
Interstate solidarity	.428***	.232***	.460***	.224***
	(.021)	(.016)	(.031)	(.018)
Institutional solidarity		.410***	.630***	.411***
		(.025)	(.037)	(.026)
Interstate solidarity × Institutional solidarity			-.093***	
			(.011)	
Czech Republic (D)				-.653***
				(.093)
Interstate solidarity × Czech Republic (D)				.186***
				(.019)
Impact on income:				
Coronavirus has not yet impacted on my income	.036*	.004	.002	.005
	(.018)	(.017)	(.017)	(.017)
Coronavirus will have no impact on my income	.097***	.060***	.059***	.060***
	(.017)	(.016)	(.016)	(.016)
Debt relief (D) (OxCGRT)	-.213***	-.198***	-.205***	-.046
	(.007)	(.006)	(.006)	(.077)
Stringency index (OxCGRT)	.006	.007	.007	.006
	(.005)	(.005)	(.005)	(.004)
Ideology	-.016	-.013	-.012	-.013
	(.013)	(.011)	(.011)	(.011)
Education:				
Secondary	.059	.013	.013	.014
	(.048)	(.048)	(.048)	(.048)
Postsecondary	.045	-.010	-.010	-.009
	(.056)	(.060)	(.060)	(.061)
Tertiary	.246***	.170**	.167**	.172**
	(.049)	(.052)	(.052)	(.052)
Employment status: Unemployed	-.033*	-.016	-.017	-.017
	(.016)	(.014)	(.014)	(.015)
Social class:				
Lower middle	.043	.060	.057	.061
	(.036)	(.033)	(.033)	(.034)
Middle class	.142***	.143***	.141***	.143***
	(.035)	(.034)	(.034)	(.035)
Upper middle class	.304***	.289***	.287***	.287***
	(.038)	(.039)	(.038)	(.039)
Higher class	.305***	.307***	.309***	.310***
	(.073)	(.075)	(.075)	(.073)
Age	-.018***	-.014**	-.014**	-.014**
	(.004)	(.004)	(.004)	(.004)

Age^2	.000***	.000**	.000**	.000**
	(.000)	(.000)	(.000)	(.000)
Country dummies	Yes	Yes	Yes	No†
Random-effects parameters:				
Variance of the level-two errors	2.04e-13	. 2.41e-13	2.39e-13	.032
	(1.07e-08)	(7.51e-13)	(7.18e-13)	(.009)
Variance of the level-one errors	.691	.607	.604	.607
	(.026)	(.020)	(.021)	(.020)
N	24,159	20,562	20,562	20,562

Notes: Reference categories: 'Coronavirus has already impacted on my personal income' (Impact on income); 'The government did not halt financial obligations for households' (Debt relief (D)); Primary (Education); 'Self-employed/employed' (Employment status); 'Working class' (Social class); 'Citizen of any other member state' (Czech Republic (D)); ***p<.001, **p<.01, *p<.05; alpha=0.5; parenthesized entries are errors clustered at the country level. † The reason for not including country dummies in this regression model is due to collinearity with the dummy for Czech Republic (1: if citizen of Czech Republic; 0: If citizen of any other member state); See the Online appendix for a detailed description of the variables.

Operationalization of variables

The dependent variable, 'the EU's image' is primarily encompassed in the Eurobarometer survey in the question: 'In general does the EU conjure up for you a very positive, fairly positive, neutral, fairly negative or very negative image?' (The variable is reversed so that high values indicate a positive image).

The main independent variable 'Interstate solidarity' is captured with the question 'How satisfied are you with the solidarity between EU member states in fighting the Corona virus pandemic?' (not at all satisfied/rather not satisfied/rather satisfied/very satisfied). The analysis also includes a set of COVID-19 related variables that are measured on both country and individual levels such as 'stringency index', 'debt relief', 'institutional solidarity' and 'impact (of COVID-19) on income'. Both the 'stringency index' and 'debt relief' are country specific variables that draw from the Oxford's COVID-19 government response tracker (OxCGRT). The 'stringency index' is an aggregate score, from 0 to 100 that OxCGRT calculated by summing up the values of different policy indicators[1] such as school closing, workplace closing, cancel public events, restrictions on public gatherings, close public transport, stay at home requirements, restrictions on internal movement, international travel controls and a variable capturing presence of public info campaigns. Incorporating the 'stringency index' in our dataset we capture the strictness of lockdown for each of the 27 countries, the date that each respondent was interviewed in the Eurobarometer data. The 'debt relief' is a dummy variable that indicates if the government halted financial obligations for households and as in the case of the 'stringency index', the variable is also country and time specific.

The Eurobarometer's question 'In general how satisfied are you with the measures taken to fight the coronavirus pandemic by the European Union' (very satisfied/ rather satisfied/ rather not satisfied/ not at all satisfied) is an important one as it distinguishes between satisfaction with institutional and interstate actions. This variable allows us to test whether the effect of satisfaction with solidarity between member states on the EU's image still holds sway when the analysis controls for a strong determinant such as satisfaction with the measures taken to fight the coronavirus pandemic by the European Union. Additionally, whether personal income has been affected by the COVID-19 pandemic can also affect one's perspective on the EU's image. Personal income is encapsulated in the Eurobarometer data with the question: 'Thinking about your personal income, which one of these statements comes close to your current situation'? The coronavirus has already impacted my personal income/coronavirus has not yet impacted on my personal income, but I expect it to in the future/coronavirus will have no impact on my personal income. The analysis lastly controls for a set of idiosyncratic variables such as ideology (from 1 to 10 where 1 stand from left and 10 for right), marital status (married or living with partner/ living alone[2]), social class (working class/ lower middle class /middle class / upper middle class / higher class), education (primary/ secondary/ post-secondary/ tertiary), gender (male/female), employment status (unemployed/self-employed or employed) and age.

The following table provides the Mean and number of cases (N) for the main variables employed in the regressions of Table A2.

[1] OxCGRT assigns a value to each indicator depending on whether an action is recommended or required to the general population or a segment of the population.

[2] Single, divorced or widowed

Table A3. Descriptive statistics.

Variables	N	Mean
EU image	26,617	3.399
Interstate solidarity	26,219	2.431
Institutional solidarity	22,282	2.511
Impact on income	26,551	2.110
Debt relief (D) (OxCGRT)	26,669	.828
Stringency index (OxCGRT)	26,629	69.602
Ideology	25,053	5.346
Education	26,633	2.893
Employment status	26,669	.435
Social class	26,370	2.582
Gender	26,669	.476
Marital status	26,544	.655
Age	26,281	50.430
Czech Republic (D)	26,669	0.039
